# Synthesis and Pharmacological Screening of Several Aroyl and Heteroaroyl Selenylacetic Acid Derivatives as Cytotoxic and Antiproliferative Agents

**DOI:** 10.3390/molecules14093313

**Published:** 2009-09-01

**Authors:** Carmen Sanmartín, Daniel Plano, Enrique Domínguez, María Font, Alfonso Calvo, Celia Prior, Ignacio Encío, Juan Antonio Palop

**Affiliations:** 1 Synthesis Section, Department of Organic and Pharmaceutical Chemistry, University of Navarra, Irunlarrea, 1, E-31008 Pamplona, Spain; 2 Molecular Modeling Section, Department of Organic and Pharmaceutical Chemistry, University of Navarra, Irunlarrea, 1, E-31008 Pamplona, Spain; 3 Oncology Division, Center for Applied Medical Research, CIMA, University of Navarra, Pío XII, 53, E-31008 Pamplona, Spain; 4 Department of Health Science, Public University of Navarra, Avda. Barañain, s/n, E-31008 Pamplona, Spain

**Keywords:** selenylacetic acid, cytotoxicity, apoptosis

## Abstract

The synthesis and cytotoxic activity of a series of twenty six aroyl and heteroaroyl selenylacetic acid derivatives of general formula Ar-CO-Se-CH_2_-COOH or Heterar-CO-Se-CH_2_-COOH are reported. The synthesis was carried out by reaction of acyl chlorides with sodium hydrogen selenide, prepared *in situ*, and this led to the formation of sodium aroylselenides that subsequently reacted with α-bromoacetic acid to produce the corresponding selenylacetic acid derivatives. All of the compounds were tested against a prostate cancer cell line (PC-3) and some of the more active compounds were assessed against a panel of four human cancer cell lines (CCRF-CEM, HTB-54, HT-29, MCF-7) and one mammary gland-derived non-malignant cell line (184B5). Some of the compounds exhibited remarkable cytotoxic and antiproliferative activities against MCF-7 and PC-3 that were higher than those of the reference compounds doxorubicin and etoposide, respectively. For example, in MCF-7 when Ar = phenyl, 3,5-dimethoxyphenyl or benzyl the TGI values were 3.69, 4.18 and 6.19 μM. On the other hand, in PC-3 these compounds showed values of 6.8, 4.0 and 2.9 μM. Furthermore, benzoylselenylacetic acid did not provoke apoptosis nor did it perturb the cell cycle in MCF-7.

## Introduction

Cancer is currently one of the leading causes of death worldwide and could become the most common cause in the future [1]. Our understanding of the biology of cancer has undoubtedly improved in the last decade. One characteristic of cancer cells is their highly proliferative nature. Consequently, inhibition of proliferative pathways is considered an effective strategy to fight cancer and a great deal of attention has recently been paid to the discovery and development of new, more selective anticancer agents. The results of numerous studies indicate that selenium is an essential trace element [2,3] with structural and enzymatic roles for viral suppression and against AIDS. This element is also implicated in delaying the aging process. Selenium deficiency has been linked to a number of disorders such as heart disease, diabetes and cancer. Selenium compounds have attracted renewed interest as chemopreventive agents for human cancer on the basis of the pioneering intervention study carried out by Clark and Combs [4]. Later epidemiological and clinical intervention studies support the protective role of selenium against the development of prostate cancer [5,6,7]. More recently, mounting evidence indicates that Se compounds inhibit neoplasm of lung, colon, mammary gland, sarcoma and glioblastoma [8,9]. In addition, selenium supplementation [10,11,12] could provide significant therapeutic potential for patients with gastrointestinal, liver and head and neck cancers. 

Furthermore, literature reports have consistently shown that the dose and chemical forms of selenium [13,14] are determinant factors in anticancer activity, with organic Se compounds being more bioavailable than the inorganic forms. 

Several chemical forms are under investigation for their potential as candidate drugs and these include methylseleno derivatives such as methylselenocysteine [15], *p*-xylylbis(methylselenide) [16] and methylseleninic acid [17], compounds with selenium in the heterocyclic ring as in selenazolidines [18], ebselen [19], D-501036 [20], BBSKE [21] or the presence of a selenide function in a symmetrical structure such as diphenyl-, dimethyl-, dibenzylselenide [22,23,24], bis(3-methyl-4-pyridyl)selenide [25] and di(3-indolyl)selenide [26].

On the other hand, the metabolism of selenium compounds is critical for activity and at least two selenium metabolite pools, hydrogen selenide and methylselenol [27,28], that induce distinct types ofantiangiogenesis, apoptosis, and cell cycle responses, have been implicated as active metabolites for the anticancer effects.

**Figure 1:**
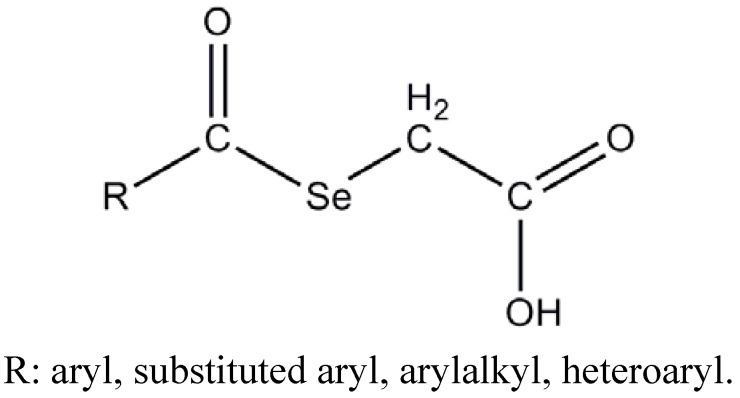
General formula of compounds.

Based on the interesting and widely observed antitumoral activity of selenium compounds and our own experience in this field [29,30], we report here the synthesis, biological evaluation and molecular modelling study of a group of selenylacetic acid derivatives **1**–**26**, (Figure 1) as potential agents in cancer therapy.

Among the strategies adopted for the design of these structures, particular consideration was given to the following approaches:

(a) The use of the selenide function due to the facile scission of Se from the organic moiety in these types of compounds. In addition, this proposed preliminary hypothesis concerning the action mechanism of these derivatives, related to the possible scission of Se from the organic moiety, allow us to determine the bond order. According to the Molecular Orbital Theory, the bond order (b.o.) is equivalent to the number of electrons in the antibonding molecular orbital minus the number of electrons in the bonding molecular orbital divided by two. This parameter can be taken as a quantitative descriptor for the bond strength and can be related to the aforementioned Se scission.
(b) The appropriate choice of the substituent on the phenyl ring was made according to a synthetic accessibility and with the aim of assessing the following aspects:
  (b.1) Influence of the substituents on the Se charge and bond stability, as well as on the acidic character of the derivatives, expressed as pKa.
  (b.2) Modulation of the electronic distribution over the aromatic moiety with a variety of electron-donating and electron-withdrawing substituents, placed at different position with respect to the lateral chain.
  (b.3) Molecular volume, conformational behaviour and hydrophobic character (expressed as AlogP), considering the presence of the keto moiety, the aromatic/heteroaromatic ring, the methylene bridge and the carboxylic moiety as the structural basic pattern.

## Results and Discussion

### Chemistry

**Scheme 1:**
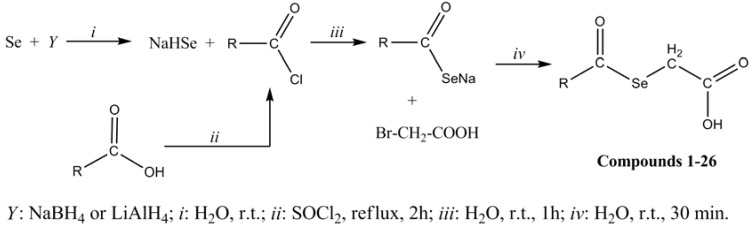
Synthesis of compounds **1**–**26**.

The synthesis of the title compounds **1**–**26** was carried out as depicted in Scheme 1. In the first step sodium hydrogen selenide was produced by reaction of powdered grey selenium with metallic hydrides in an appropriate medium. In the second step the aroyl or heteroaroyl chloride was reacted with sodium hydrogen selenide to produce the intermediate sodium aroyl selenides. The last step involved the formation of selenylacetic acids **1**–**26** by treatment of this intermediate with α-bromoacetic acid. This synthesis was based on a published procedure with modifications [31] following our own protocol.

The compounds were obtained in yields from 3 to 78%. The analytical data are shown in Table 1. The purity levels of compounds **1**–**26** were assessed by TLC and elemental analyses and their structures were identified from spectroscopic data (IR, ^1^H NMR). The IR spectra of these compounds revealed intense absorption bands in the ranges 1,720–1,700 cm^–1^ and 1,690–1,665 cm^–1^, assigned to carboxyl and carbonyl groups, respectively. The frequencies of these absorptions varied according to the substitution pattern on the arylidene ring. As expected, a decrease in the carbonyl absorption frequencies was observed for compounds having electron-donating groups such as methylenedioxy, trimethoxy or *tert*-butyl attached to the benzylidene ring in comparison to electron-withdrawing groups (chloro, bromo).

### Biological Evaluation

#### Cytotoxic activity in PC-3

Initially, the new compounds **1**–**26** were evaluated for their *in vitro* cytotoxic activity against a human prostate cancer cell line (PC-3, ATCC, Manassas, VA, USA) using the MTT assay [32]. We selected this cell line because there are numerous clinical trials that show the activity of selenium compounds in the reduction of prostate cancer [5,9,15,16]. The results are tabulated as IC_50_ values. All experiments were independently performed at least three times and the values were calculated after 72 hours exposure (drug concentrations of 2, 5, 7 and 10 μM). The results are shown in Table 2. 

The maximum cytotoxic activity was exhibited by compounds **1**, **2**, **11**, **14** and **21**. By comparing the activities of compounds with those of methylseleninic acid and etoposide, both effective drugs against prostate cancer that have been employed as controls for cytotoxic assays, the results revealed that three of the new compounds −**1**, **11**, **14** − were more active than methylseleninic acid and etoposide and that five were better than etoposide. It is noteworthy that the introduction of a new methoxy groups in the 4-position in **14** to give **15** results in suppression of the antitumour activity on the PC-3 cell line. These findings, along with the observed antitumour activity of compound **14**, suggest that the activity of the compounds depends on the location of the methoxy substituents on the phenyl ring. With the aim of evaluating the preliminary structure-activity relationships to gain an understanding of the activity shown by such compounds as antitumorals, we carried out a molecular modelling study on the aforementioned derivatives.

**Table 1:** Physical constants for compounds **1**–**26**.

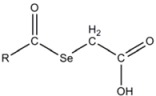						
Ref.	R	Yield (%)	M. p. (ºC)	Recryst. Solvent	CHN	Anal (%) Calcd/Found
**1**	phenyl	75	83^a^	Toluene	C_9_H_8_O_3_Se	C, 44.44/44.47; H, 3.29/3.24; N, 0.00/0.00.
**2**	4-cyanophenyl	78	147^a^	Toluene	C_10_H_7_NO_3_Se	C, 44.77/44.85; H, 2.61/2.67; N, 5.22/4.98.
**3**	4-(trifluoromethyl)phenyl	62	108^a^	Toluene	C_10_H_7_F_3_O_3_Se	C, 38.58/38.73; H, 2.25/2.15; N, 0.00/0.00.
**4**	4-chlorophenyl	19	135-136^b,c^	Toluene	C_9_H_7_ClO_3_Se	C, 38.95/38.71; H, 2.54/2.43; N, 0.00/0.00.
**5**	4-methylphenyl	53	92^a^	Toluene	C_10_H_10_0_3_Se	C, 44.69/44.95; H, 3.89/3.89; N, 0.00/0.00.
**6**	4-( *tert*-butyl)phenyl	19	99-103^b,c^	Chloroform	C_13_H_16_O_3_Se	C, 52.18/52.04; H, 5.39/5.31; N, 0.00/0.05.
**7**	4-methoxyphenyl	16	104-107^b^	Chloroform/Carbon tetrachloride	C_10_H_10_O_4_Se	C, 43.97/43.49; H, 3.69/3.52; N, 0.00/0.00.
**8**	2-chlorophenyl	44	123-125^b^	Carbon tetrachloride	C_9_H_7_ClO_3_Se	C, 38.95/38.97; H, 2.54/2.52; N, 0.00/0.06.
**9**	2-bromophenyl	32	124-128^b^	Carbon tetrachloride	C_9_H_7_BrO_3_Se	C, 33.57/33.14; H, 2.19/2.02; N, 0.00/0.20.
**10**	2-iodophenyl	3	105-108^b^	Carbon tetrachloride	C_9_H_7_IO_3_Se	C, 29.29/29.14; H, 1.91/1.92; N, 0.00/0.05.
**11**	benzyl	48	74^a^	Toluene	C_9_H_10_O_3_Se	C, 46.69/46.87; H, 3.89/3.89; N, 0.00/0.00.
**12**	2-phenylethyl	11	65-69^b^	Hexane	C_11_H_12_O_3_S ¼ H_2_O	C, 47.93/48.06; H, 4.57/4.47; N, 0.00/0.18.
**13**	3,5-dichlorophenyl	17	108-109^b^	Carbon tetrachloride	C_9_H_6_Cl_2_O_3_Se·½ H_2_O	C, 33.67/33.56; H, 2.20/1.84; N, 0.00/0.06.
**14**	3,5-dimethoxyphenyl	69	117^a^	Toluene	C_11_H_12_O_5_Se	C, 43.56/43.70; H, 3.96/3.88; N, 0.00/0.00.
**15**	3,4,5-trimethoxyphenyl	14	107-110^b^	Ethanol	C_12_H_14_O_6_Se	C, 43.26/43.32; H, 4.24/3.97; N, 0.00/0.11.
**16**	3,4-methylendioxyphenyl	47	106-113^b^	Carbon tetrachloride	C_10_H_8_O_5_Se	C, 41.83/41.56; H, 2.81/2.67; N, 0.00/0.02.
**17**	naphthyl	37	130^a^	Toluene	C_13_H_10_O_3_Se	C, 53.24/52.99; H, 3.41/3.25; N, 0.00/0.00.
**18**	diphenylmethyl	16	127-130^b^	Carbon tetrachloride	C_16_H_14_O_3_Se	C, 56.90/56.88; H, 4.33/4.11; N, 0.00/0.14.
**19**	4-pyridyl	11	119-121^b,c^	Ether / hexane	C_8_H_7_NO_3_Se	C, 39.36/39.39; H, 2.89/2.78; N, 5.74/5.67.
**20**	3-pyridyl	15	147-150^b,c^	Methanol	C_8_H_7_NO_3_Se	C, 39.36/39.57; H, 2.89/2.76; N,5.74/5.63.
**21**	3-(2-chloropyridyl)		157^a^	Toluene	C_8_H_6_ClNO_3_Se	C, 34.47/34.68; H, 2.15/2.17; N, 5.01/5.26.
**22**	3-(2-propylthiopyridyl)	36	109-111^b^	Carbon tetrachloride	C_11_H_13_NO_3_SSe	C, 41.51/41.28; H, 4.12/3.90; N, 4.40/4.24.
**23**	2-thienyl	33	82-84^b^	Carbon tetrachloride	C_7_H_6_O_3_SSe	C, 33.75/33.45; H, 2.43/2.54; N, 0.00/0.07.
**24**	pyrazinyl	10	138-140^b^	Isopropanol	C_7_H_6_N_2_O_3_Se	C, 34.30/34.18; H, 2.47/2.49; N, 1.43/1.37.
**25**	2-quinolyl	6	131-132^b,c^	Toluene	C_12_H_9_NO_3_Se	C, 49.00/49.33; H, 3.08/3.13; N, 4.76/4.75.
**26**	3-quinolyl	9	187-189^b,c^	Chloroform	C_12_H_9_NO_3_Se	C, 49.00/48.95; H, 3.08/3.23; N, 4.76/4.66.

^a ^Determined by differential scanning calorimetry; ^b^ Determined by thermomicroscopy; ^c ^Fusion with degradation.

**Table 2:** Cytotoxic activities of compounds **1**–**26** against the PC-3 cell line.

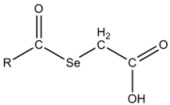		
Compound	R	PC-3 cell line
		IC_50_ (μM)
**1**	phenyl	6.8
**2**	4-cyanophenyl	10.0
**3**	4-(trifluoromethyl)phenyl	NE^a^
**4**	4-chlorophenyl	NE
**5**	4-methylphenyl	NE
**6**	4-*tert*-butylphenyl	NE
**7**	4-methoxyphenyl	NE
**8**	2-chlorophenyl	NE
**9**	2-bromophenyl	NE
**10**	2-iodophenyl	NE
**11**	benzyl	2.9
**12**	2-phenylethyl	NE
**13**	3,5-dichlorophenyl	NE
**14**	3,5-dimethoxyphenyl	4.0
**15**	3,4,5-trimethoxyphenyl	NE
**16**	3,4-methylenedioxyphenyl	NE
**17**	naphthyl	NE
**18**	diphenylmethyl	NE
**19**	4-pyridyl	NE
**20**	3-pyridyl	NE
**21**	3-(2-chloropyridyl)	10.0
**22**	3-(2-propylthiopyridyl)	NE
**23**	2-thienyl	NE
**24**	pyrazinyl	NE
**25**	2-quinolyl	NE
**26**	3-quinolyl	NE
**MSA^b^**		8.38 [33]
**Etoposide**		13.6 ± 2.2 [34]

^a ^NE = no effect is observed; ^b^ methylseleninic acid.

### Molecular modelling

**Figure 2:**
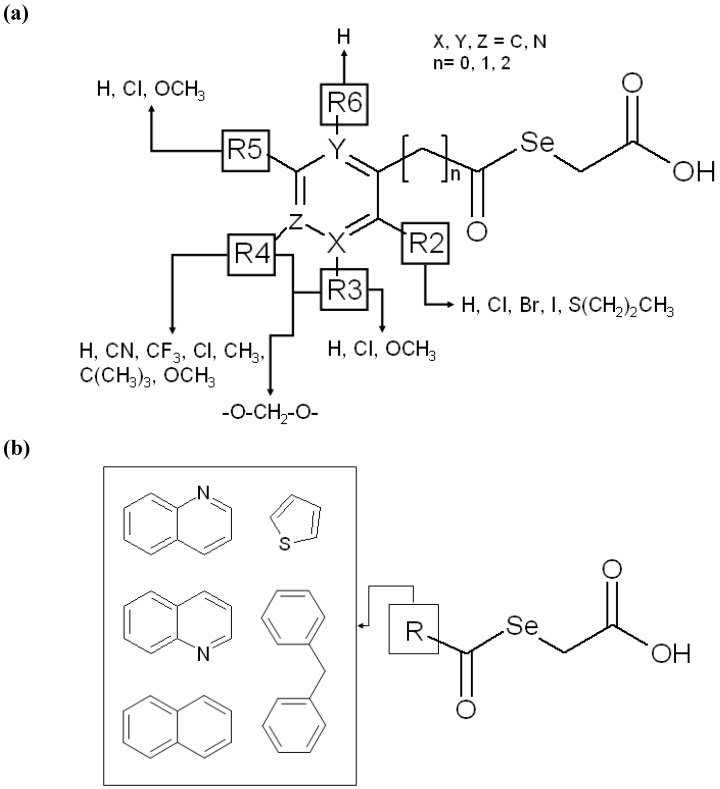
Overview of structural variations carried out for the studied derivatives: (a) aryl or heteroaryl monocyclic derivatives, (b) aryl or heteroaryl bicyclic derivatives.

From the molecular modelling point of view, three approaches were applied in an effort to gain an insight into the structural requirements for activity in these compounds (Figure 2).

Firstly, it was planned to obtain some descriptive parameter at a molecular level, such as the log of the partition coefficient expressed as ALogP and the molecular volume, particularly the volume of the aromatic moiety, that would allow discrimination between active and inactive compounds.

A second approach was carried out. This was based on the pharmacophoric hypothesis that an active compound can be characterised by a set of chemical functions with a certain spatial arrangement and molecular shape that could be related to the observed biological activity but would not be associated with the inactive compounds. Thus, the presence of certain chemical functions is not only necessary, but these functions must be exposed in a manner that can be recognised by the associated binding site — a property that requires specific conformational behaviour [35]. In agreement with this approach, a compound can be considered as a collection of multiple instances, one for each conformation. An active compound contains at least one active instance, while an inactive compound does not contain any active instance. On the assumption that all of the active compounds bind to the same site, we would expect their binding conformations to have similar shapes. For this second approach it is necessary to carry out a study of the conformational behaviour of the compounds and subsequently select and analyse the supposed bioactive conformations of the compounds.

The third approach considered here concerned the structural characteristics of designed molecules, especially the presence of the selenium atom, the proposed structural variations related to the length of the chain, which acts like a bridge between the keto group and the aromatic or heteroaromatic moiety, and the electronic characteristics of the groups located in these rings. These structural variations led us to carry out a study from the mechano-quantic point of view in order to obtain data on parameters such as the value of the partial charge on the selenium, the energy and distribution of the HOMO and LUMO orbitals as well as the electronic density (ED) and molecular electrostatic potential (MEP) distribution. The aim was to try to relate these values with the biological activity of our derivatives. 

The calculations were performed on a Dell Precision 380 workstation, provided with the software package Discovery Studio v1.7 [36], and on a SGI Virtu VS100 workstation, provided with MOPAC2009 [37] and Mercury [38] software packages. 

Once the models for the compounds had been constructed, the initial geometries were fully minimized to an energy gradient below 10^–3^ kcal mol^–1^Å^–1^. The minimum energy conformers were superimposed, with the ring moiety taken as adjusting atoms. The effectiveness of the superimposed models was evaluated in terms of the Root Mean Square (RMS) values obtained. The energy differences between the different conformations analysed for each trajectory were in the range 2–5 kcal. 

Analysis of the resulting data (Table 3, Table 4) enabled the following general observations to be made in an attempt to correlate the data with the cytotoxic activity levels found for the compounds on the PC-3 cell line.

**Figure 3:**
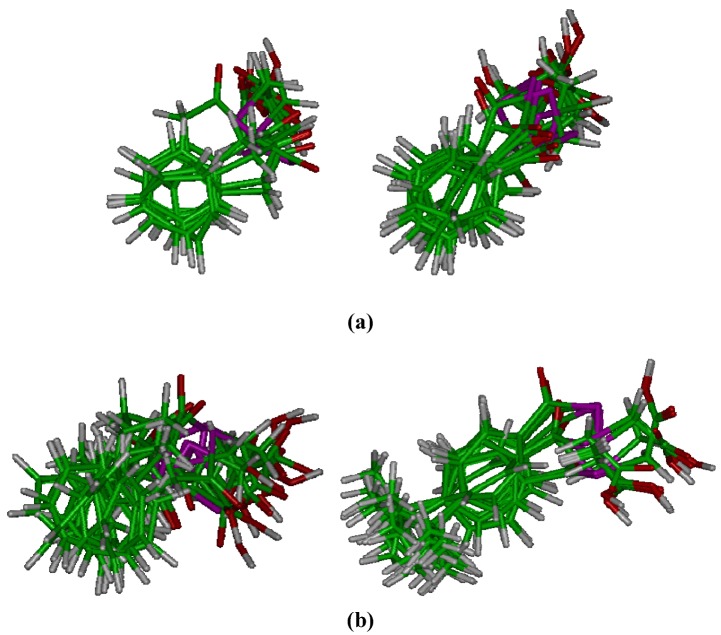
Conformational models for some representative compounds: (a) active compounds, left **11**; right **1**; (b) inactive compounds: left **12**; right **6**. Carbon in green; hydrogen in grey; oxygen in red; selenium in violet.

1. The permissible maximum volume for the aromatic fragment is approximately 111–112 Å^3^. Thus, the bicyclic compounds (naphthyl, diphenylmethyl and quinolyl derivatives) and the monocyclic derivatives with voluminous substituents are inactive.

2. In relation to the conformational behaviour, it can be observed that, in general, in the conformational trajectory of active compounds the number of extended conformations is greater than that in the inactive ones, with a better general superposition of the conformations, and the total volume of the conformational trajectory is also greater (rms data not shown for the sake of brevity). An example is shown in Figure 3.

3. With respect to the ALogP98 values determined on the preliminary geometries obtained after the first minimization (Table 3), although a direct correlation could not be established, the resulting data did shed some light on the biological data, since the active products have a value between 1.518 (compound **21**, IC_50 _= 10.0 μM) and 1.829 (compound **11**, the most active compound, IC_50 _= 2.9 μM). As expected, some structural modifications give rise to a marked reduction in the AlogP values, as is the case for compounds **19** and **20**, derived from pyridine, with a value of 0.644, or for compound **24**, derived from pyrazine, with a negative value for this parameter. On the other hand, it can be seen that the compounds with an AlogP greater than 1.90 are inactive in this assay.

**Table 3:** Molecular descriptors obtained for the analyzed compounds (aryl or heteroaryl monocyclic derivatives) ^a^.

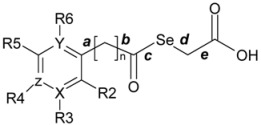													
Ref.	X	Y	Z	n	R_2_	R_3_	R_4_	R_5_	R_6_	AlogP	Vol^b^	μ (D)^c^	IC_50_(μM)^e^
**1**	C	C	C	0	H	H	H	H	H	1.795	68.58	3.465	6.8
**2**	C	C	C	0	H	H	CN	H	H	1.673	82.16	2.865	10.0
**3**	C	C	C	0	H	H	CF_3_	H	H	2.737	92.64	1.137	NE
**4**	C	C	C	0	H	H	Cl	H	H	2.459	83.19	2.628	NE
5	C	C	C	0	H	H	CH_3_	H	H	2.281	82.76	4.430	NE
6	C	C	C	0	H	H	C(CH_3_)_3_	H	H	3.195	125.55	4.584	NE
**7**	C	C	C	0	H	H	OCH_3_	H	H	1.778	89.51	5.337	NE
**8**	C	C	C	0	Cl	H	H	H	H	2.459	82.97	2.874	NE
**9**	C	C	C	0	Br	H	H	H	H	2.543	91.02	4.032	NE
**10**	C	C	C	0	I	H	H	H	H	2.373	100.49	3.259	NE
**11**	C	C	C	1	H	H	H	H	H	1.829	82.73	3.418	2.9
**Ref.**	**X**	**Y**	**Z**	**n**	**R_2_**	**R_3_**	**R_4_**	**R_5_**	**R_6_**	**AlogP**	**Vol^b^**	**μ (** **D)^c^**	**IC_50_(** **μ** **M)^d^**
**12**	C	C	C	2	H	H	H	H	H	2.286	93.29	4.243	NE
**13**	C	C	C	0	H	Cl	H	Cl	H	3.123	98.15	2.441	NE
**14**	C	C	C	0	H	OCH_3_	H	OCH_3_	H	1.762	111.05	4.591	4.0
**15**	C	C	C	0	H	OCH_3_	OCH_3_	OCH_3_	H	1.745	131.49	5.090	NE
**16**	C	C	C	0	H	-O-CH_2_-O-		H	H	1.563	88.78	4.352	NE
**19**	C	C	N	0	H	H	-	H	H	0.644	64.45	1.081	NE
**20**	N	C	C	0	H	H	H	H	H	0.644	64.64	3.543	NE
**21**	N	C	C	0	Cl	H	H	H	H	1.518	78.98	4.094	10.0
**22**	N	C	C	0	S(CH_2_)_2_CH_3_	H	H	H	H	2.597	126.66	4.964	NE
**23**	2-thienyl									1.520	64.02	3.315	NE
**24**	N	N	C	0	H	H	H	H	-	-0.078	60.35	3.096	NE
**MSA**	-	-	-	-	-	-	-	-	-	-	-	-	8.38
**Etoposide**	-	-	-	-	-	-	-	-	-	-	-	-	13.6 ± 2.2

^a^ General structure for the analysed compounds showing the bonds (*a*–*e*) selected for the conformational analysis. ^b^ Volume (average value obtained from the lowest energy conformations) of the cyclic fragment in Å^ 3^. ^c^ Dipolar moment (in Debyes) calculated for the representative low-energy. ^d^ Cytotoxic activity in PC-3 cell line, NE= no effect.

**Table 4:** Molecular descriptors obtained for the analyzed compounds (aryl or heteroaryl bicyclic derivatives) ^a^.

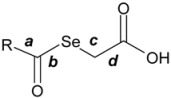					
Ref.	R	AlogP	Vol^b^	μ (D)^c^	IC_50_(μM)^d^
**17**	naphthyl	2.703	106.65	3.759	NE
**18**	diphenylmethyl	3.324	144.34	3.632	NE
**25**	2-quinolyl	2.409	102.41	4.451	NE
**26**	3-quinolyl	1.981	102.22	2.685	NE
**MSA**	-	-	-	-	8.38
**Etoposide**	-	-	-	-	13.6 ± 2.2

^a^ General structure for the analysed compounds showing the bonds (*a*–*d*) selected for the conformational analysis. ^b ^Volume (average value obtained from the lowest energy conformations) of the cyclic fragment in Å^ 3^. ^c^ Dipolar moment (in Debyes) calculated for the representative low-energy conformation. ^d^ Cytotoxic activity in PC-3 cell line, NE= no effect.

**Figure 4:**
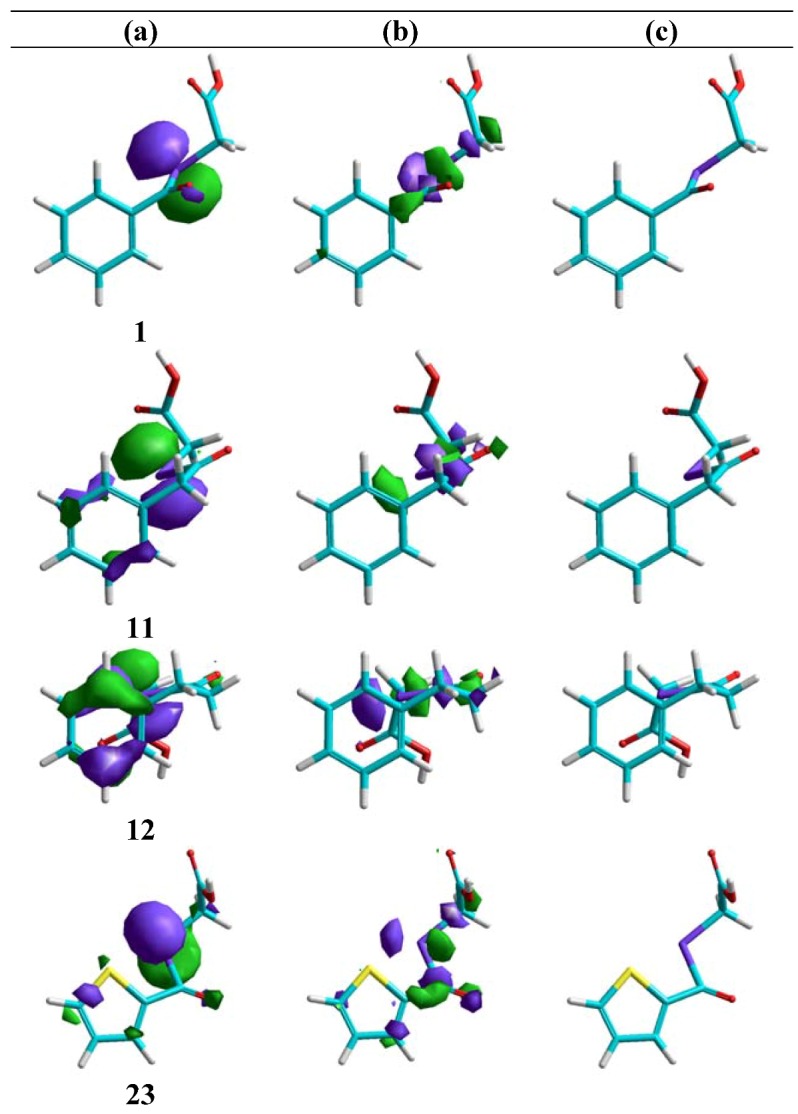
Influence of the alkyl chain and ring on the HOMO 0 (a), LUMO 0 (b) distribution, valuated as descriptive parameters, showed on a low-energy representative conformation (c). Carbon in cyan; hydrogen in white; oxygen in red; selenium in violet; sulphur in yellow.

**Figure 5:**
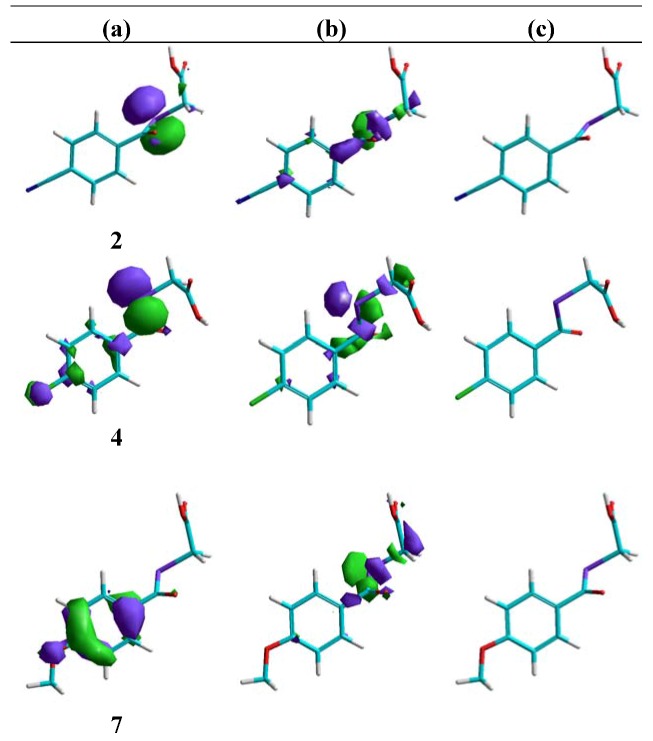
Influence of the substituents placed in 4-position on the HOMO 0 (a), LUMO 0 (b) distribution, valuated as descriptive parameters, showed on a low-energy representative conformation (c). Carbon in cyan; hydrogen in white; oxygen in red; selenium in violet; nitrogen in blue; chlorine in green.

4. With respect to the length of the bridging chain between the aromatic moiety and the keto group, the introduction of a methylene fragment significantly improves the activity (IC_50 _= 6.8 μM for compound **1** and IC_50 _= 2.9 μM for compound **11**; Table 3 and Figure 4c). However, the introduction of a second methylene leads to the disappearance of the activity. This change could be related to the conformational behaviour. For example, in compound **12** the preferred conformations are the folded ones (see Figure 4c).

5. As regards the dipolar moment, a correlation could not be established and great variability was observed for this parameter, even within the conformational trajectory of each individual compound.

6. With respect to the descriptor parameters obtained by quantum chemistry semi-empirical calculations, the structural variations that lead to a negative value for the partial charge on the selenium lead to the loss of activity. Concerning the distribution of the frontier molecular orbitals (see 4a, 4b, 5a and 5b), it was also found that in the most active compounds the HOMO orbital is preferentially located on the selenium, with a significant contribution from carbons of the aromatic nucleus. In general it can be observed that the presence of substituents in the 4-position brings about the displacement of the HOMO towards the ring, with the contribution of selenium decreasing along with the activity. As an example, the distribution of this orbital for compounds **1** (IC_50 _= 6.8 μM, Figure 4**a**) and **2** (IC_50 _= 10.0 μM, Figure 5**a**) can be compared with that of compound **7**, which contains a methoxy group and is inactive. Nevertheless a correlation with the data of energy for orbital HOMO and LUMO, nor with the gap (LUMO-HOMO energy) cannot be established (Table 5, Table 6). 

**Table 5:** Mechano-quantic descriptive parameters (semiempirical: PM6) obtained for the analyzed compounds (aryl or heteroaryl monocyclic derivatives).

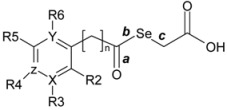																		
Ref.	X	Y	Z	n	R_2_	R_3_	R_4_	R_5_	R_6_	pKa^b^	Bond order			HOMO^c^	LUMO^d^	E_L-M_	Q_Se^e^	IC_50_
											*a*	*b*	*c*					(M)^f^
**1**	C	C	C	0	H	H	H	H	H	2,119	1,8315	0,9068	0,9618	-9,210	-1,552	7,658	0.0577	6.8
**2**	C	C	C	0	H	H	CN	H	H	2,057	1,8478	0,9050	0,9608	-9,422	-2,022	7,400	0.0778	10.0
**3**	C	C	C	0	H	H	CF_3_	H	H	2,148	1,8492	0,9048	0,9608	-9,399	-1,894	7,505	-0.0303	NE
**4**	C	C	C	0	H	H	Cl	H	H	2,188	1,7914	0,9659	0,9624	-9,382	-1,666	7,716	-0.0394	NE
**5**	C	C	C	0	H	H	CH_3_	H	H	2,184	1,8230	0,9037	0,9613	-9,002	-1,487	7,515	0.0491	NE
**6**	C	C	C	0	H	H	C(CH_3_)_3_	H	H	2,182	1,8256	0,9027	0,9611	-9,108	-1,403	7,705	0.0485	NE
**7**	C	C	C	0	H	H	OCH_3_	H	H	2,196	1,8159	0,8940	0,9592	-9,039	-1,208	7,831	0.0433	NE
**8**	C	C	C	0	Cl	H	H	H	H	2,360	1,8508	0,9320	0,9673	-9,258	-1,384	7,874	0.0292	NE
**9**	C	C	C	0	Br	H	H	H	H	2,346	1,8611	0,9172	0,9524	-9,295	-1,244	8,051	0.0134	NE
**10**	C	C	C	0	I	H	H	H	H	2,388	1,8721	0,9069	0,9626	-8,864	-1,582	7,282	-0.0182	NE
**11**	C	C	C	1	H	H	H	H	H	2,370	1,8419	0,9742	0,9841	-9,439	-0,936	8,503	0.0681	2.9
**12**	C	C	C	2	H	H	H	H	H	2,378	1,8360	0,9799	0,9622	-9,485	-1,138	8,347	0.0580	NE
**13**	C	C	C	0	H	Cl	H	Cl	H	1,858	1,8482	0,9123	0,9626	-9,410	-1,961	7,449	-0.0331	NE
**14**	C	C	C	0	H	OCH_3_	H	OCH_3_	H	2,791	1,9205	0,9126	0,9673	-9,275	-1,063	8,212	0.0693	4.0
**15**	C	C	C	0	H	OCH_3_	OCH_3_	OCH_3_	H	2,431	1,8021	0,9528	0,9633	-8,770	-1,329	7,441	0.0652	NE
**16**	C	C	C	0	H	-OCH_2_O-		H	H	2,083	1,8413	0,9068	0,9620	-9,223	-1,630	7,593	0.0720	NE
**19**	C	C	N	0	H	H	-	H	H	1,985	1,7891	0,9918	0,9766	-9,546	-1,717	7,829	0.0825	NE
**20**	N	C	C	0	H	H	H	H	H	2,230	1,7775	0,9840	0,9767	-9,483	-1,663	7,820	0.0712	NE
**21**	N	C	C	0	Cl	H	H	H	H	2,145	1,8279	0,9540	0,9686	-9,368	-1,712	7,656	0.0129	10.0
**22**	N	C	C	0	S(CH_2_)_2_CH_3_	H	H	H	H	2,486	1,8722	0,8969	0,9628	-8,871	-1,585	7,286	-0.0093	NE
**23**					2-thienyl					2,181	1,7872	0,9417	0,9598	-9,204	-1,696	7,508	-0.0438	NE
**24**	N	N	C	0	H	H	H	H	-	1,813	1,7829	1,0108	0,9746	-9,406	-2,030	7,376	0.1126	NE

**Table 6:** Mechano-quantic descriptive parameters (semiempirical: PM6) obtained for the analyzed compounds (aryl or heteroaryl bicyclic derivatives) ^a^.

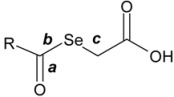										
Ref.	R	pKa^b^	Bond order			HOMO^c^	LUMO^d^	ΔE_L-M_	Q_Se^e^	IC_50_
			a	b	c					(μM)^f^
**17**	Naphtyl	2,655	1,8432	0,8921	0,9578	-9,076	-1,647	7,429	0.0644	NE
**18**	Diphenylmethyl	2,592	1,8829	0,9492	0,9689	-9,456	-1,110	8,346	0.0598	NE
**25**	2-quinolyl	2,306	1,7884	1,0012	0,9606	-9,214	-1,918	7,296	0.0918	NE
**26**	3-quinolyl	2,375	1,8453	0,8972	0,9589	-9,222	-1,881	7,341	0.0619	NE

*Notes for* Table 5*,* Table 6: ^a^ General structure for the analyzed compounds showing the bonds (*a*-*b*) selected for the bond order determination. ^b^ pKa value calculated for the low-energy representative conformation ^c^ HOMO 0 orbital energy, in eV, calculated for the low-energy representative conformation ^d^ LUMO 0 orbital energy, in eV, calculated for the low-energy representative conformation ^e^ Charge on Selenium atom, value obtained for the low-energy representative conformation. ^f^Cytotoxic activity in PC-3 cell line, NE= no effect.

7. With respect to the bond order (Table 5, Table 6) a correlation with the activity cannot be established, which contradicts our starting hypothesis related to the stability of the C-Se bond. 

8. In relation to the acidic character evaluated by the pKa values (Table 5, Table 6), the values of pKa for the most active compounds oscillate between 2.05 and 2.80, although the proposed structural modifications apparently do not affect in a significant manner this parameter.

In the present state of our research on these selenium derivatives, we do not try to propose a pharmacophore applying only a conformacional analysis. We have carried out a conformacional analysis in order to obtain data about the conformacional behavior of these molecules, with a high degree of conformational freedom, and to sample what are the preferred conformations, between that we hoped to find the bioactive one.

### Cytotoxic activity in CCRF-CEM, HTB-54, HT-29, MCF-7 and 184B5

In order to investigate the effect of the active compounds in more detail, we examined the activity on proliferation in other cancer cell lines. The most active compounds in PC-3 were tested, along with the reference compound doxorubicin, for cytotoxic and antiproliferative activities. Tests were carried out in the Department of Health Science, Public University of Navarra against a panel of four human tumour cell lines: lung (HTB-54), colon (HT-29), leukaemia (CCRF-CEM) and breast adenocarcinoma (MCF-7). Cytotoxicity assays were performed based on the reactivity of MTT [3-(4,5-dimethylthiazol-2-yl)-2,5-diphenyl-tetrazolium bromide], as described by NCI [39]. The results are expressed as GI_50_ values, *i.e.,* the concentration that reduces by 50% the growth of treated cells with respect to untreated controls, TGI, the dose that completely inhibits cell growth, and LD_50_, the concentration that kills 50% of the cells. The cytotoxic effect of each substance was tested at five different doses between 0.01 and 100 μM, or at lower levels when the GI_50_ was less than 10 nM. Mean GI_50_, TGI, and LD_50_ values are summarized in Table 7. Doxorubicin was used as a control. As guidance with regard to selectivity, all of the compounds were further examined for toxicity in a mammary gland cell culture derived from non-malignant cells (184B5). Drug concentrations ranged from 0.01 to 100 μM.

**Table 7:** Cytotoxic activities (average GI_50_^a^, TGI^b^ and LD_50_^c^ values) for compounds against tumour cell lines.

Comp.	C.P^a^ (μM)	Cell lines				
		CCRF-CEM	HTB-54	HT-29	MCF-7	184B5
**1**	**^b^GI_50_**	>100	0.58	7.39	0.09	1.97
	**^c^TGI**	>100	42.47	55.98	3.69	22.50
	**^d^LD_50_**	>100	>100	>100	58.01	76.73
**2**	**GI_50_**	>100	1.90	8.95	2.64	2.29
	**TGI**	>100	9.91	51.95	6.54	20.14
	**LD_50_**	>100	>100	>100	23.12	78.91
**11**	**GI_50_**	>100	11.14	16.19	0.006	1.05
	**TGI**	>100	>100	72.19	4.18	9.64
	**LD_50_**	>100	>100	>100	53.49	72.34
**14**	**GI_50_**	>100	16.82	5.99	1.57	0.0009
	**TGI**	>100	>100	47.42	6.19	8.21
	**LD_50_**	>100	>100	>100	52.65	62.55
**21**	**GI_50_**	>100	11.79	7.53	0.003	0.05
	**TGI**	>100	>100	60.94	8.27	1.89
	**LD_50_**	>100	>100	>100	89.21	7.20
**Doxorub.**	**GI_50_**	0.033	<0.01	nd^e^	0.88	nd
	**TGI**	0.071	1.25	nd	>100	nd
	**LD_50_**	0.29	3.45	nd	>100	nd

^a^ Cytotoxic parameters;^ b^ Dose that inhibits 50% of cell growth; ^c^ Dose that completely inhibits cell growth; ^d^ Dose that kills 50% of cells; ^e^ n.d.: not determined.

The data show that the compounds under investigation influenced tumour cell growth differently depending on the cell line. The human breast adenocarcinoma cell line (MCF-7) was most sensitive to the antiproliferative effects of the investigated compounds. In particular, **1** (GI_50_ = 0.09 μM), **11** (GI_50_ = 0.006 μM), **14** (GI_50_ = 1.57 μM ) and **21** (GI_50_ = 0.003 μM) were strongly antiproliferative. Compounds **1**, **11** and **21** were 10, 147 and 293 times more active, respectively, than standard doxorubicin (GI_50_ = 0.88 μM). Besides, if we compare the TGI values for these compounds (TGI = 3.69, 4.18, 6.19 and 8.27 μM, respectively), all of them are lower than that of doxorubicin. In addition, **11** and **21** exhibited a better antitumoral profile than paclitaxel (GI_50_ = 0.010 ± 0.04 and TGI = 20 ± 1.9 μM) and vinorelbine (GI_50_ = 5 ± 1.3 and TGI = 100 ± 10.2 μM), both drugs that are widely used in clinic. The least sensitive cell line was CCRF-CEM leukemia with cytotoxic parameters >100 μM for all the compounds. HT-29 and HTB-54 showed moderate levels of susceptibility to the compounds with the exceptions of **1** (GI_50_ = 7.39 and 0.58 μM) and **2** (GI_50_ = 8.95 and 1.90 μM). 

**Figure 6:**
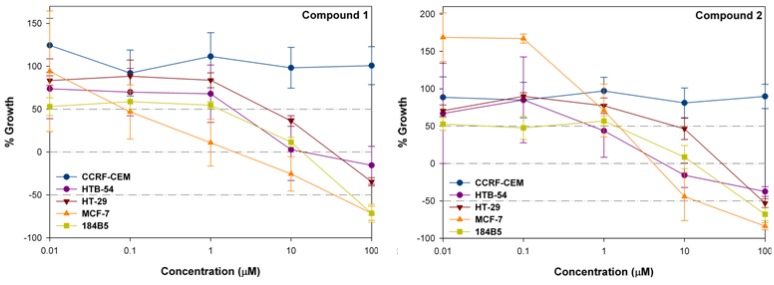
Cytotoxic effects of **1** and **2** on CCRF-CEM, HTB-54, HT-29, MCF-7 and 184B5 cells. Cells were incubated in the presence of each compound at the indicated concentration for 72 hours. Cytotoxicity was then determined by a colorimetric microassay based on the use of MTT. Data are expressed as percentage growth ± SEM from at least 3 independent experiments performed in quadruplicate.

As an example, curves with the original data from which the GI_50_, TGI and LD_50_ values for compounds **1** and **2** were calculated are shown in Figure 6. Fortunately, the LD_50_ in 184B5 is higher than in MCF-7 and the selectivity indexes are 1.3 and 3.4, respectively (184B5/MCF-7 LD_50_ ratio).

It is evident that compound **1** displayed the most prominent antiproliferative activity against all treated cell lines compared to the untreated control. This compound is therefore a representative candidate for these compounds for preliminary studies in order to explore a possible mechanism of action.

### Apoptosis

Mounting evidence indicates that apoptosis is a critical mechanism for cancer prevention by Se compounds [40,41]. For this reason, we investigated whether apoptosis was involved in cell growth inhibition in the MCF-7 cells by **1**.

**Figure 7:**
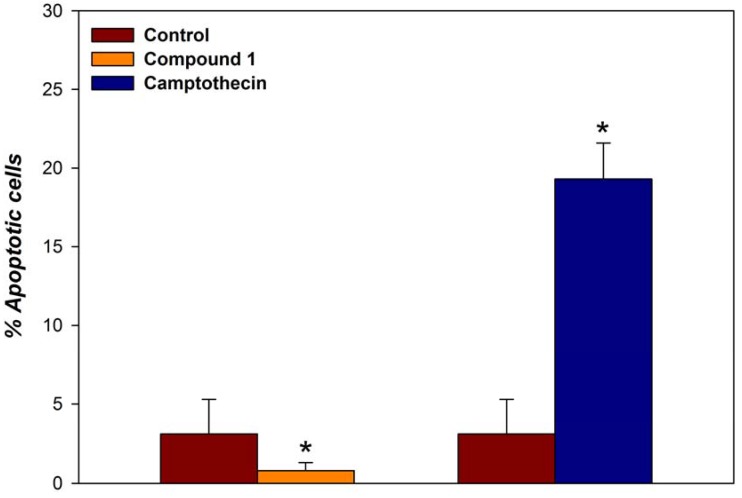
MCF-7 cells were incubated either with 25 µM of the indicated compound or vehicle (control cells) for 48 hours. The results are presented as the mean ± SEM of three independent experiments (duplicate wells). * p < 0.01 with respect to the control.

The apoptotic status of the cells after 48 hours of treatment with 25 μM of the corresponding compound was determined using the Apo-Direct kit (BD Pharmingen) [42] based on the TUNEL technique. Camptothecin was used as a positive control. The results obtained are shown in Figure 7. As can be seen, for compound **1** the induction of cell death was independent of the apoptotic process.

*Effects on cell cycle progression*

**Figure 8:**
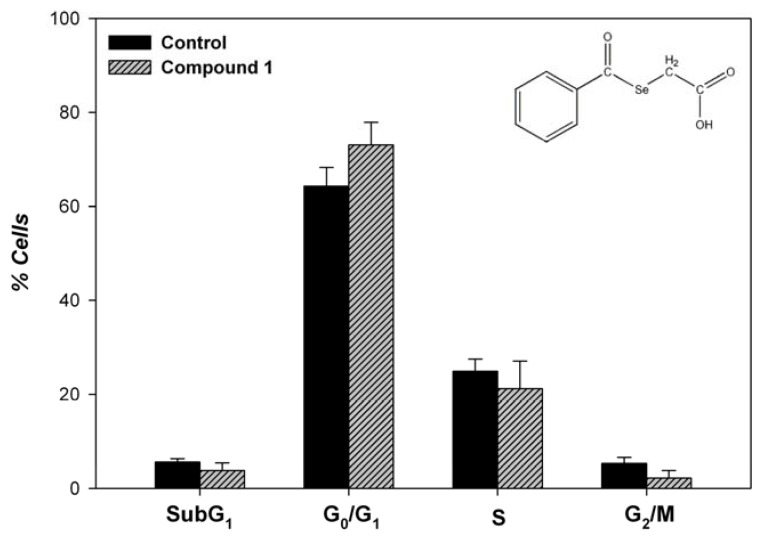
Cell cycle phase distribution of MCF-7 cells after 48 hours of treatment with 25 μM of the indicated compound or vehicle (control). Results are expressed as percentages of total cell counts. Each bar represents mean ± SEM of three independent experiments (duplicate wells).

Cell cycle arrest is one of the targets of many anticancer drugs, including doxorubicin and camptothecin. In an effort to ascertain whether compound **1** could affect cell cycle progression in MCF-7 cells, these cells were treated with 25 μM of the corresponding compound for 48 hours and cell cycle progression was determined by flow cytometry analysis [42]. As shown in Figure 8, DNA flow cytometric analysis indicated that treatment of the cells with compound 1 did not induce any specific phase arrest of the cell cycle.

## Conclusions

In summary, we have synthesized a series of 26 aroyl and heteroaroyl selenylacetic acids and evaluated the cytotoxicity against a prostate cancer cell line (PC-3). Five compounds (**1**, **2**, **11**, **14** and **21**) showed interesting activity and three of them (**1**, **11** and **14**) were more powerful than etoposide and methylseleninic acid. Exploration of the SAR study suggested that the permissible maximum volume for the aromatic fragment is approximately 111–112 Å. A study of the ALogP data indicated that the compounds with values much greater than 1.90 were inactive. In terms of the length of the bridging chain between the aromatic moiety and the keto group, the presence of a methylene fragment improves the activity. We also tested some selected compounds against a panel of four human tumoral cell lines (CCRF-CEM, HTB-54, HT-29, MCF-7) and one mammary gland-derived non-malignant cell line (184B5). Compounds **1**, **11** and **21** exhibited excellent growth inhibition activity against MCF-7, with values in the nanomolar range, and moderate values in HTB-54 and HT-29. However, these compounds did not show inhibitory potency against CCRF-CEM. Comparable activity was observed on two of the cell lines studied, one a hormone-independent prostate (PC-3) and the other a hormone-dependent MCF-7 breast cancer cell line. This could suggest that the mode of action is to a large extent unrelated to the expression of the androgen receptor (PC-3 negative) and oestrogen receptor (MCF-7 positive). Compound **1**, which exhibited the best profile, was examined as an apoptotic agent and cell cycle modulator in MCF-7. The preliminary results revealed that the mechanism of action is independent of apoptosis and additional studies are required to determine the mechanism by which this compound exerts its activity. This work is currently in progress in our laboratory. The results reported here open up new perspectives for future investigations into the synthesis of new compounds of this family and these are potentially useful for the modulation of pharmacological potential with enhanced and selective anticancer activity. 

## Experimental

### General

Melting points were determined by differential scanning calorimetry with a Perkin-Elmer DSC Diamond and by thermomicroscopy with a Mettler FP82+FP80 apparatus (Greifense, Switzerland) and have not been corrected. The ^1^H NMR spectra (Table 8) were recorded on a Bruker 400 Ultrashield^TM^ (Rheinstetten, Germany), using TMS as the internal standard (Table 8). The IR spectra (Table 8) were performed on a Thermo Nicolet FT-IR Nexus in KBr pellets (Table 8). Elemental microanalyses were carried out on vacuum-dried samples (Table 1) using an Elemental Analyser (LECO, CHN-900 Elemental Analyser). Silica gel 60 (0.040–0.063 mm) 1.09385.2500 (Merck KGaA, 64271 Darmstadt, Germany) was used for Column Chromatography and Alugram^®^ SIL G/UV_254_ (Layer: 0.2 mm) (Macherey-Nagel GmbH & Co. KG. Postfach 101352. D-52313 Düren, Germany) was used for Thin Layer Chromatography. Chemicals were purchased from E. Merck (Darmstadt, Germany), Scharlau (F.E.R.O.S.A., Barcelona, Spain), Panreac Química S.A. (Montcada i Reixac, Barcelona, Spain), Sigma-Aldrich Química, S.A. (Alcobendas, Madrid, Spain), Acros Organics (Janssen Pharmaceuticalaan 3a, 2440 Geel, België), Maybridge (Cambridge, CB5 8BZ, UK) and Lancaster (Bischheim-Strasbourg, France).

**Table 8:** Spectroscopic data for compounds **1**–**26**.

Ref.	IR (KBr, υ in cm^-1^)	^1^H NMR (400 MHz, δ/ppm, *J* in Hz)
**1**	1702, 1678	DMSO*-d_6_*, 3.81 (s, 2H, Se-CH_2_-COOH), 7.59 (m, 2H, H_3_ + H_5_), 7.75 (tt, 1H, H_4_, *J*_4-5_=*J*_4-3_=7.9, *J*_4-6_=*J*_4-2_=1.2), 7.90 (dd, 2H, H_2_ + H_6_, *J*_6-5_=*J*_2-3_=8.4), 12.80 (br s, 1H, COOH).
**2**	2232, 1681	CDCl_3_, 3.91 (s, 2H, Se-CH_2_-COOH), 7.81 (d, 2H, H_3_ + H_5_, *J*_3-2_=*J*_5-6_=8.7), 8.00 (d, 2H, H_2_ + H_6_).
**3**	1694, 1712	CDCl_3_ 3.91 (s, 2H, Se-CH_2_-COOH), 7.78 (dd, 2H, H_3_+ H_5_, *J*_3-2_=*J*_5-6_=8.1, *J*_3-CF3_=*J*_5-CF3_=0.5), 8.02 (dd, 2H, H_2_ + H_6_, *J*_2-CF3_=*J*_6-CF3_=0.6).
**4**	1697, 1686	DMSO*-d_6_*, 3.81 (s, 2H, Se-CH_2_-COOH), 7.66 (d, 2H, H_3_+H_5_; *J*_3-2_= *J*_5-6_=8.5), 7.91 (d, 2H, H_2_+H_6_), 12.89 (br s, 1H, COOH).
**5**	1691	CDCl_3_, 2.43 (s, 3H, CH_3_), 3.85 (s, 2H, Se-CH_2_-COOH), 7.29 (d, 2H, H_3_+ H_5, _ *J*_3-2_=*J*_5-6_= 8.1), 7.81 (d, 2H, H_2_ + H_6_).
**6**	1715, 1666	CDCl_3_, 1.36 (s, 9H, C-(CH_3_)_3_), 3.85 (s, 2H, Se-CH_2_-COOH), 7.51 (d, 2H, H_3_+H_5_, *J*_3-2_= *J*_5-6_=8.7), 7.86 (d, 2H, H_2_+H_6_).
**7**	1707, 1692	CDCl_3_, 3.83 (s, 2H, Se-CH_2_-COOH), 3.91 (s, 3H, CH_3_O), 6.97 (d, 2H, H_3_+H_5_, *J*_3-2_= 9.0), 7.90 (d, 2H, H_2_+H_6_).
**8**	1707, 1688	CDCl_3_, 3.89 (s, 2H, Se-CH_2_-COOH), 7.37-7.43 (m, 1H, H_5_), 7.49-7.50 (m, 2H, H_4_+H_3_), 7.76-7.79 (m, 1H, H_6_).
**9**	1703, 1684	CDCl_3_, 3.89 (s, 2H, Se-CH_2_-COOH), 7.40 (tt, 1H, H_5_, *J*_5-6_=1.8, *J*_5-4_=7.5 *J*_5-3_=8.9), 7.44 (tt, 1H, H_4_, *J*_4-3_=1.4 Hz, *J*_4-6_=7.6), 7.70 (dd, 1H, H_3_), 7.72 (dd, 1H, H_6_).
**10**	1701, 1677	CDCl_3_, 3.90 (s, 2H, Se-CH_2_-COOH), 7.23 (dt, 1H, H_5_, *J*_5-3_=1.5, *J*_5-6_=7.9, *J*_5-4_=7.7), 7.48 (dt, 1H, H_4_,*J*_4-6_=0.4, *J*_4-3_=7.7), 7.69 (dd, 1H, H_3_); 8.00 (dd, 1H, H_6_).
**11**	1694, 1685	CDCl_3_, 3.62 (s, 2H, Se-CH_2_-COOH), 3.90 (s, 2H, Ar-CH_2_-COSe), 7.32 (m, 2H, H_3_ + H_5_), 7.38 (m, 3H, H_2_ + H_4_ + H_6_).
**12**	1708, 1686	CDCl_3_, 3.03 (s, 4H, Ar-CH_2_-CH_2_-COSe + Ar-CH_2_-CH_2_-COSe), 3.69 (s, 2H, Se-CH_2_-COOH), 7.20-7.26 (m, 3H, H_3_+H_4_+H_5_), 7.30-7.34 (m, 2H, H_2_+H_6_).
**13**	1699, 1667	CDCl_3_, 3.84 (s, 2H, Se-CH_2_-COOH), 7.57 (s, 1H, H_4_), 7.70 (s, 2H, H_2_+H_6_), 8.44 (br s, 1H, COOH).
**14**	1714, 1696	CDCl_3_, 3.85 - 3.86 (s + s, 8H, 2 OCH_3_ + Se-CH_2_-COOH, *J*_CH2-Se_=72.2), 6.72 (dt, 1H, H_4_, *J*_4-2_=*J*_4-6_=0.8 Hz, *J*_4-OCH3_=2.3), 7.04 (dd, 2H, H_2_ + H_6_, *J*_2-OCH3_*=J*_6-OCH3_=2.2).
**15**	1703, 1671	CDCl_3_, 3.86 (s, 2H, Se-CH_2_-COOH), 3.94 (s, 6H, 3,5-diCH_3_O), 3.95 (s, 3H, 4-CH_3_O), 7.16 (s, 2H, H_2_).
**16**	1701, 1675	DMSO*-d_6_*, 3.76 (s, 2H, Se-CH_2_-COOH), 6.18 (s, 2H, O-CH_2_-O), 7.09 (dd, 1H, H_5_, *J*_5-2_= 0.9 Hz, *J*_5-6_=7.8), 7.34 (dd, 1H, H_2_, *J*_2-6_=2.9), 7.55 (dd, 1H, H_6_), 12.79 (br s, 1H, COOH).
**17**	1719, 1671	CDCl_3_, 3.92 (s, 2H, Se-CH_2_-COOH), 4.40 (br s, 1H, COOH), 7.63 (m, 2H, H_4_+ H_5_), 7.91 (m, 3H, H_3_ + H_6_ + H_7_), 8.00 (d, 1H, H_8_, *J*_8-7_=8.1), 8.48 (s, 1H, H_2_).
**18**	1720, 1693	CDCl_3,_ 3.68 (s, 2H, Se-CH_2_-COOH), 5.23 (s, 1H, (Ph)_2_-CH-COSe), 7.31-7.40 (m, 10 H, 2H_2_, 2H_6_, 2H_3_, 2H_5_, 2 H_4_).
**19**	1717, 1659	DMSO*–d_6_*, 3,87 (s, 2H, Se-CH_2_-COOH), 7,78 (td, 2H, H_3_+H_5_), 8,85 (td, 2H, H_2_+H_6_).
**20**	1713, 1673	DMSO*-d_6_*, 3.87 (s, 2H, Se-CH_2_-COOH), 7.61-7.65 (m, 1H, H_5_), 8.26-8.30 (m, 1H, H_4_), 8.87-8.90 (m, 1H, H_6_), 9.02-9.03 (m, 1H, H_2_), 12.84 (br s, 1H, COOH).
**21**	1724, 1690	DMSO-*d_6_*, 3.87 (s, 2H, Se-CH_2_-COOH), 7.63 (ddd, 1H, H_5_, *J*_5-4_=7.7, *J*_5-6_=4.8, *J*_5-Cl_= 1.1), 8.24 (ddd, 1H, H_4_, *J*_4-Cl_*=*1.1), 8.63 (ddd, 1H, H_6_, *J*_6-Cl_=1.1), 12.90 (br s, 1H, COOH).
**22**	1700, 1668	DMSO-*d_6_*_,_ 0.97 (t, 3H, CH_3_), 1.58-1.70 (m, 2H, S-CH_2_-CH_2_-CH_3_), 3.11 (dt, 2H, S-CH_2_-CH_2_-CH_3_), 3.81 (s, 2H, Se-CH_2_-COOH), 7.32 (dd, 1H, H_4_, *J*_4-5_=4.8, *J*_4-6_=7.8), 7.97 (dt, 1H, H_5_, *J*_5-6_=1.7), 8.29 (dd, 1H, H_6_), 12.89 (br s, 1H, COOH).
**23**	1712, 1643	DMSO*-d_6_*_,_ 3.80 (s, 2H, Se-CH_2_-COOH), 7.30 (dd, 1H, H_4_, *J*_4-5_=3.9 Hz,*J*_4-3_=4.9 Hz), 8.02 (dd, 1H, H_5_, *J*_5-3_=1.1 Hz), 8.16 (dd, 1H, H_3_), 12.82 (bs, 1H, COOH).
**24**	1703, 1674	DMSO*-d_6_*, 3.74 (s, 2H, Se-CH_2_-COOH), 8.87 (dd, 1H, H_6_, *J*_6-3_=1.2 Hz, *J*_6-5_=2.4 Hz), 9.05 (dd, 1H, H_5_, *J*_5-3_=0.2 Hz), 9.06 (dd, 1H, H_3_), 12.73 (br s, 1H, COOH).
**25**	1714, 1690	DMSO*-d_6_*, 3.73 (s, 2H, Se-CH_2_-COOH), 7.82 (t, 1H, H_7_, *J*_7-6_=7.5, *J*_7-8_=8.0), 7.94 (t, 1H, H_6_, *J*_6-5_=8.3), 7.97 (d, 1H, H_5_), 8.15 (d, 1H, H_8_), 8.19 (d, 1H, H_3_, *J*_3-4_=8.4), 8.66 (d, 1H, H_4_), 12.68 (br s, 1H, COOH).
**26**	1709, 1675	DMSO*-d_6_,* 3,91 (s, 2H, Se-CH_2_-COOH), 7,77 (t, 1H, H_6_, *J*_6-7_=7,6 ), 7,98 (t, 1H, H_7_), 8,13 (d, 1H, H_5_, *J*_5-8_=8,3), 8,31 (d, 1H, H_8_), 9,07 (d, 1H, H_4_, *J*_4-2_=1,8), 9,23 (d, 1H, H_2_),12,75 (bs, 1H, COOH).

### General procedure for the preparation of selenylacetic acids ***1**–**6**, **8**–**26***

A solution of sodium borohydride (1.00 g, 26.3 mmol) in distilled water (12.5 mL) was added to a stirred suspension of grey selenium (1.00 g, 12.6 mmol) in distilled water (12.5 mL) at room tempera-ture. The reaction mixture was stirred until an almost colourless solution of NaHSe was formed. The acyl chloride (12.6 mmol) was added in small portions and the reaction mixture was magnetically stirred for 1 hour. A yellow solution was formed and the bromoacetic acid (1.75 g, 12.6 mmol) was added. Within 30 minutes a solid was formed. The product was filtered off and washed with distilled water (3 × 25 mL). The products were recrystallised from an appropriate solvent (Table 1). The following compounds were synthesized according to this procedure: *(Benzoylselenyl)acetic acid* (**1**). *(4-cyanobenzoylselenyl)acetic acid* (**2**). *(4-trifluoromethylbenzoylselenyl)acetic acid* (**3**). *(4-chlorobenzoylselenyl)acetic acid* (**4**). *(4-methylbenzoylselenyl)acetic acid* (**5**). *(4-*tert*-butylbenzoyl-selenyl)acetic acid* (**6**). *(2-chlorobenzoylselenyl)acetic acid* (**8**). *(2-bromobenzoylselenyl)acetic acid* (**9**). *(2-iodobenzoylselenyl)acetic acid* (**10**). *Phenylacetoylselenylacetic acid* (**11**). *(3-phenyl-propanonylselenyl)acetic acid* (**12**). *(3,5-dichlorobenzoylselenyl)acetic acid* (**13**). *(3,5-dimethoxy-benzoylselenyl)acetic acid* (**14**). *(3,4,5-trimethoxybenzoylselenyl)acetic acid* (**15**). *(3,4-methylenedioxy-benzoylselenyl) acetic acid* (**16**). *(2-naphthoylselenyl)acetic acid* (**17**). *(diphenylacetylselenyl)acetic acid* (**18**). *(4-pyridoylselenyl)acetic acid* (**19**). *(3-pyridoylselenyl)acetic acid* (**20**). *(3-(2-chloro)-pyridoylselenyl)acetic acid* (**21**). *(3-(2-propylthio)pyridoylselenyl)acetic acid* (**22**). *(2-thienoylselenyl)-acetic acid* (**23**). *(pyrazinoylselenyl)acetic acid* (**24**). *(2-quinoloylselenyl)acetic acid* (**25**). *(3-quinoloylselenyl)acetic acid* (**26**).

### Procedure for the preparation of (4-methoxyphenylselenyl)acetic acid *(**7**)*

An equivalent of grey-powder selenium was suspended in tetrahydrofuran (20 mL) and two equivalents of lithium aluminium hydride were added slowly. The carbonyl chloride was added to the reaction mixture and the mixture was stirred for 1 h. The suspension was filtered and the solid was discarded. An equivalent of bromoacetic acid was added to the liquid filtrate. After 20 min the mixture was poured onto 500 g of ice. The resulting solid was filtered off, washed and recrystallised (Table 1).

### Cytotoxic activity in PC-3

PC-3 cells were seeded in 96-well plates (Millipore, Eschborn, Germany) at a density of 5 × 10^3^ cells per well. The samples were incubated at 37 ºC under 5% CO_2_ overnight prior to the addition of the compounds. Compounds were diluted in complete medium. After 3 days of incubation, 10 μL MTT solution (5 mg/mL in PBS) was added to the cells in each well and these were stored for an additional 4 h at 37 ºC. The absorbance of formazan at *λ* = 570 nm was measured on a *Polarstar Galaxy plate reader* (BMG LabTechnologies GmbH, Offenburg, Germany). The percentage of viable cells was calculated to obtain IC_50_-values.

PC-3 are human tumorigenic and metastatic prostate cancer cells and these were obtained from American Type Culture Collection (ATCC, Manassas, VA, USA) (passage 36). The cells were cultured under standard conditions (Dulbecco’s RPMI 1640 medium, with GlutamaxTM 1, Invitrogen supplemented with 10% fetal bovine serum, Fetalclone III, SH30109.03, HYCLONE and 1% Penicillin-Streptomycin, Invitrogen, Carlsbad, CA, USA). 

### Molecular modelling

The initial computational work was performed on a Dell Precision 380 workstation, provided with the software package Discovery Studio v1.7. The three-dimensional models of the studied compounds were constructed, in the vacuum phase, using atoms and structural fragments from the Viewer module (Discovery Studio) and using the Dreiding force field [43]. Once the models were constructed, a preliminary conformational analysis was carried out. The applied protocol (Diverse Conformational Generation integrated in the Pharmacophore protocol Discovery Studio) can be summed up as follows: (a) Initial construction of the model and first minimization by application of the Dreiding minimize protocol (Steepest descent algorithm with a convergence criterion of 10^–6^). The AlogP98 [44,45] descriptor (an implementation of the atom-based ALogP method) was calculated for each compound. (b) Application of the BEST routine for conformation generation (First: Conjugate-gradient minimization in torsion space; second: conjugate-gradient minimization in Cartesian space; third: Quasi_Newton minimization in Cartesian space). (c) Elimination of those conformations whose relative energy is greater than 10 Kcal/mol at a global minimum. (d) Analysis of conformational trajectory and selection of representative lowest energy conformations. Root mean square (rms) deviations of the structures were monitored. The energy differences between the different conformations analysed for each trajectory were in the range 2–5 Kcal. 

For each of the compounds, ten lowest energy conformations were selected and a new minimization cycle was applied. The volumes of the ring moiety and the whole molecule were calculated for each of the new representative low-energy conformations selected.

The mechano-quantic analysis of the conformations obtained in the previous step was carried out with the package *Mopac2009*, PM6 [46] (or PM3 [47] for halogen and/or sulfur-containing derivatives) semi-empirical approaches, with the geometry optimized using an eigenvector following algorithm. The energy and distribution of the HOMO and LUMO orbitals, the selenium charge, the electronic density (ED) and molecular electrostatic potential (MEP) distribution and the Dipolar moment were obtained for each of the conformations obtained. The data corresponding to the representative lowest energy conformation for each compound was selected and used in the establishment of the preliminary structure-activity relationships.

### Cytotoxic activity in CCRF-CEM, HTB-54, HT-29, MCF-7 and 184B5

The cytotoxic effects of each substance were tested at five different doses between 0.01 and 100 μM. Each substance was initially dissolved in DMSO at a concentration of 0.1 M, and serial dilutions were prepared using culture medium. The plates with cells from the different lines, to which medium containing the substance under test were added, were incubated for 72 h at 37 °C in a humidified atmosphere containing 5% CO_2_. European Collection of Cell Cultures (ECACC) or American Type Culture Collection (ATCC) provided human tumour cell lines. Four cell lines were used: one human lymphocytic leukemia (CCRF-CEM) and three human solid tumours, one colon carcinoma (HT-29), one lung carcinoma (HTB-54) and one breast adenocarcinoma (MCF-7). CCRF-CEM, HT-29 and HTB-54 cells were grown in RPMI 1640 medium (Life Technologies, Carlsbad, CA, USA) supplemented with 10% foetal calf serum, 2 mM L-glutamine, 100 units/mL penicillin, 100 μg/mL streptomycin and 10 mM HEPES buffer (pH = 7.4). MCF-7 cells were grown in EMEM medium (Clonetics) supplemented with 10% foetal calf serum, 2 mM L-glutamine, 100 units/mL penicillin and 100 μg/mL streptomycin. 184B5 cells were grown Hams F-12/DMEM (50:50) supplemented as described by Li et al. [48]. Cytotoxicity was then determined by the MTT method. Results are expressed as GI_50_ values, the concentration that reduces by 50% the growth of treated cells with respect to untreated controls, TGI, the dose that completely inhibits cell growth, and LD_50_, the concentration that kills 50% of the cells. Data were obtained from at least three independent experiments performed in quadruplicate.

### Apoptosis and cell cycle

For breast adenocarcinoma MCF-7 cells, the apoptotic status and cell cycle analysis of the cells were determined using the *Apo-Direct* kit (BD Pharmingen), based on the TUNEL technique, under the conditions described by the manufacturer. 
